# National seroepidemiological study of COVID‐19 after the initial rollout of vaccines: Before and at the peak of the Omicron‐dominant period in Japan

**DOI:** 10.1111/irv.13094

**Published:** 2023-02-01

**Authors:** Takeshi Arashiro, Satoru Arai, Ryo Kinoshita, Kanako Otani, Sho Miyamoto, Daisuke Yoneoka, Taro Kamigaki, Hiromizu Takahashi, Hiromi Hibino, Mai Okuyama, Ai Hayashi, Fuka Kikuchi, Saeko Morino, Sayaka Takanashi, Takaji Wakita, Keiko Tanaka‐Taya, Tadaki Suzuki, Motoi Suzuki

**Affiliations:** ^1^ Center for Surveillance, Immunization, and Epidemiologic Research National Institute of Infectious Diseases Tokyo Japan; ^2^ Department of Pathology National Institute of Infectious Diseases Tokyo Japan; ^3^ Infectious Disease Emergency Specialist (IDES) Training Program Ministry of Health, Labour and Welfare Tokyo Japan; ^4^ National Institute of Infectious Diseases Tokyo Japan

**Keywords:** coronavirus disease 2019 (COVID‐19), humoral immunity, Japan, seroepidemiologic studies, severe acute respiratory syndrome coronavirus 2 (SARS‐CoV‐2), vaccines

## Abstract

**Background:**

Based on routine surveillance data, Japan has been affected much less by COVID‐19 compared with other countries. To validate this, we aimed to estimate SARS‐CoV‐2 seroprevalence and examine sociodemographic factors associated with cumulative infection in Japan.

**Methods:**

A population‐based serial cross‐sectional seroepidemiological investigation was conducted in five prefectures in December 2021 (pre‐Omicron) and February–March 2022 (Omicron [BA.1/BA.2]‐peak). Anti‐nucleocapsid and anti‐spike antibodies were measured to detect infection‐induced and vaccine/infection‐induced antibodies, respectively. Logistic regression was used to identify associations between various factors and past infection.

**Results:**

Among 16 296 participants (median age: 53 [43–64] years), overall prevalence of infection‐induced antibodies was 2.2% (95% CI: 1.9–2.5%) in December 2021 and 3.5% (95% CI: 3.1–3.9%) in February–March 2022. Factors associated with past infection included those residing in urban prefectures (Tokyo: aOR 3.37 [95% CI: 2.31–4.91], Osaka: aOR 3.23 [95% CI: 2.17–4.80]), older age groups (60s: aOR 0.47 [95% CI 0.29–0.74], 70s: aOR 0.41 [95% CI 0.24–0.70]), being vaccinated (twice: aOR 0.41 [95% CI: 0.28–0.61], three times: aOR 0.21 [95% CI: 0.12–0.36]), individuals engaged in occupations such as long‐term care workers (aOR: 3.13 [95% CI: 1.47–6.66]), childcare workers (aOR: 3.63 [95% CI: 1.60–8.24]), food service workers (aOR: 3.09 [95% CI: 1.50–6.35]), and history of household contact (aOR: 26.4 [95% CI: 20.0–34.8]) or non‐household contact (aOR: 5.21 [95% CI:3.80–7.14]) in February–March 2022. Almost all vaccinated individuals (15 670/15 681) acquired binding antibodies with higher titers among booster dose recipients.

**Conclusions:**

Before Omicron, the cumulative burden was >10 times lower in Japan (2.2%) compared with the US (33%), the UK (25%), or global estimates (45%), but most developed antibodies owing to vaccination.

## 1 INTRODUCTION

Coronavirus disease 2019 (COVID‐19), caused by severe acute respiratory syndrome coronavirus 2 (SARS‐CoV‐2), has resulted in substantial morbidity and mortality globally.^1^ Because SARS‐CoV‐2 infection can be asymptomatic and some infections are missed due to underdiagnosis and underreporting, a seroprevalence study can complement routine surveillance to provide a better picture of the cumulative burden of COVID‐19. Also, coupling with questionnaires regarding various exposures can elucidate sociodemographic factors associated with cumulative infection.

**FIGURE 1:**
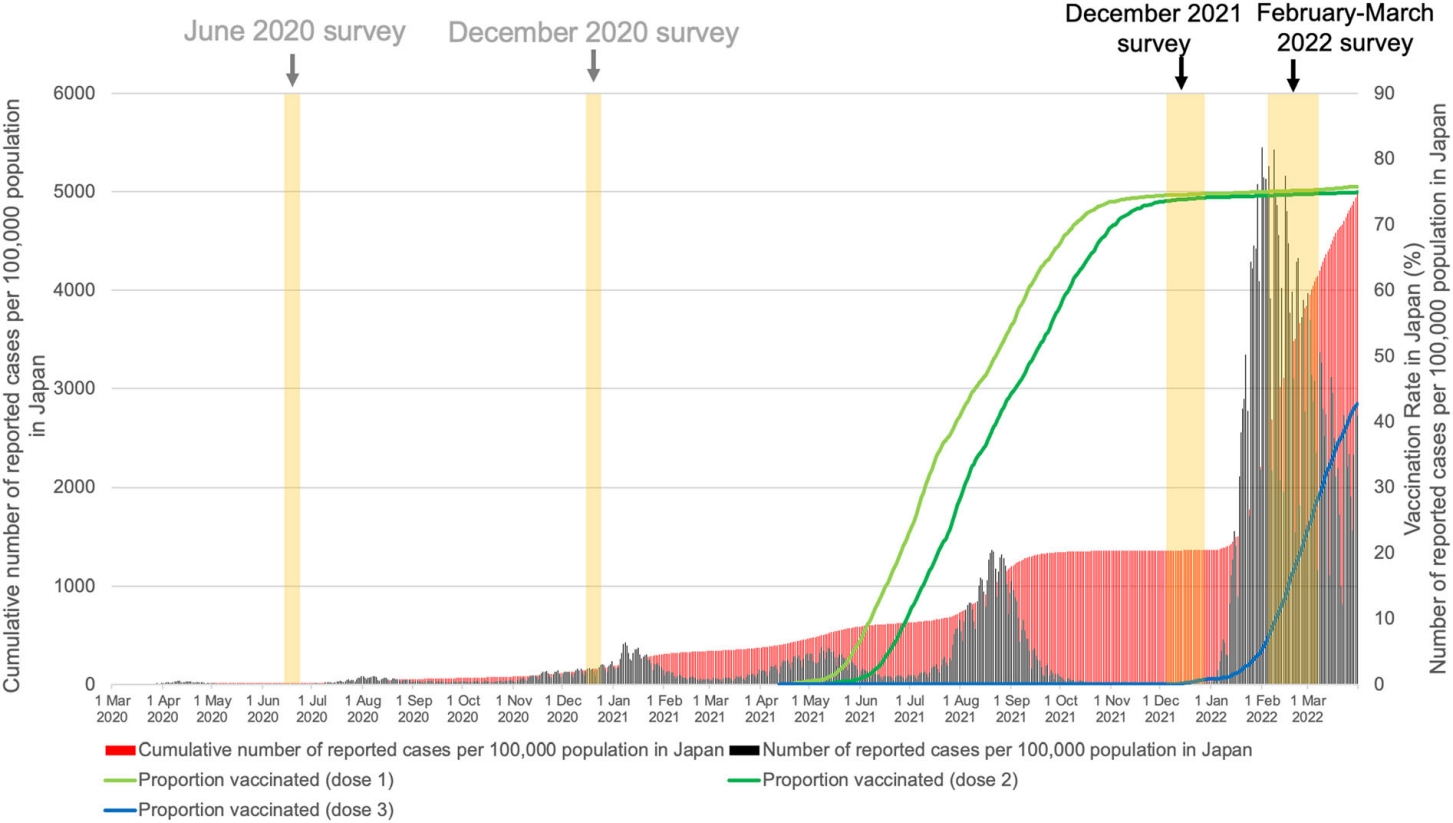
Number of reported COVID‐19 cases since the beginning of the pandemic and COVID‐19 vaccine rollout in Japan (data sources: Ministry of Health, Labour and Welfare, Japan [https://www.mhlw.go.jp/stf/covid‐19/open‐data.html] and Digital Agency, Japan [https://info.vrs.digital.go.jp/dashboard])

Japan has experienced six waves of COVID‐19 epidemics as of March 2022, with the first three waves due to the original SARS‐CoV‐2 strain, and the fourth, fifth, and sixth waves by its Alpha, Delta, and Omicron variants respectively (Figure 1). Population‐based seroprevalence surveys have been conducted twice by the Ministry of Health, Labour and Welfare and the Research Institute of Tuberculosis since the beginning of the COVID‐19 pandemic. Although these surveys were conducted partly via convenient sampling, even in Tokyo with one of the highest reported cases (both absolute numbers and per population) consistently throughout the pandemic, the seroprevalence was extremely low in June 2020 (0.1%) after the first wave, and December 2020 (1.4%) at the beginning of the third wave.^2^, ^3^

The rollout of the primary series of COVID‐19 vaccines started in mid‐February 2021. BNT162b2, mRNA‐1273, and AZD1222 have been approved for use (with minimal use of AZD1222).^4^ The primary series (doses 1 and 2) followed manufacturer‐recommended intervals. The rollout of primary series stabilized by November 2021 at around 75% and the rollout of the mRNA booster (third) dose was initiated in December 2021 (individuals became eligible for their booster 6–7 months after completing the primary series). Despite the increasing cumulative burden and rapid vaccine rollout, seroepidemiological studies at the national level had not been performed. Therefore, the Ministry of Health, Labour and Welfare, Japan, and the National Institute of Infectious Diseases (NIID) conducted a national seroepidemiological investigation with two objectives: (1) To examine the cumulative burden of SARS‐CoV‐2 infection in urban and rural settings with an exploration of sociodemographic factors associated with past infection and (2) to assess the extent of antibody development among vaccinated individuals with a description of vaccinees who did not develop detectable antibodies. The investigation was conducted through two surveys, one in December 2021 (before the Omicron‐dominant period) and the other in February–March 2022 (peak of the Omicron‐dominant period).

## 2 METHODS

### 2.1 Survey design and participants

Using the Basic Resident Register, residents of Miyagi, Tokyo, Aichi, Osaka, and Fukuoka prefectures were randomly sampled via multi‐stage sampling (Figure S1). Four prefectures (Tokyo, Aichi, Osaka, and Fukuoka) were chosen to represent urban areas with a high burden of SARS‐CoV‐2 infection, and Miyagi prefecture was selected to represent a rural area with a low burden. For each prefecture, at least one municipality from each of the following three municipality types was chosen for the surveys: Small (<100 000 population), medium (≥100 000 population), and large (ordinance‐designated city/special ward). Samples were split to the relative population sizes of the municipality, and residents were randomly sampled from each of these municipalities. We planned to enroll a total of 30 000 individuals for two surveys in five prefectures (3000 individuals per prefecture per survey). Assuming a response rate of 40%, we randomly sampled 75 000 individuals aged 20 years or older and invited them via mail. Only one participant was allowed from each household. Individuals who agreed to participate in the study answered a self‐administered questionnaire and came to the designated site, where they provided written consent and had their blood drawn. Different participants were included in the two surveys without overlap. Briefly, the questionnaire included general information (age, sex, occupation, etc.), comorbidities, COVID‐19 vaccination status, history of household and non‐household close contact, and history of past SARS‐CoV‐2 infection. In planning the investigation, we referred to the “Population‐based age‐stratified seroepidemiological investigation protocol for COVID‐19 infection” published by the World Health Organization (WHO),^5^ and the study was considered “Unity‐aligned” based on the criteria previously described.^6^

### 2.2 Serology

We utilized two widely used commercial electrochemiluminescence immunoassays to detect vaccine‐induced and infection‐induced antibodies; one was Elecsys Anti‐SARS‐CoV‐2 (Roche), which detects binding antibodies against the nucleocapsid protein (anti‐N antibodies), and the other was Elecsys Anti‐SARS‐CoV‐2 S (Roche), which detects antibodies against the receptor‐binding domain of the ancestral strain spike protein (anti‐spike [S] antibodies). Because COVID‐19 vaccines approved in Japan code only for the spike protein, anti‐N antibodies are induced by infection but not by vaccines, where anti‐S antibodies are induced by both infection and vaccines. A cutoff index (COI) of 1.0 and a cutoff value of 0.8 U/ml, as determined by the manufacturer, were used to determine the presence/absence of anti‐N antibodies and anti‐S antibodies, respectively. With these cutoffs, assays have adequate sensitivity (N: 99.4% against PCR‐positive samples 20+ days post‐symptom onset and S: 97.7% against PCR‐positive samples 14+ days post‐symptom onset) and specificity (N: 98.1% and S: 100% both against PCR‐negative samples) as per external evaluation by Public Health England (United Kingdom Health Security Agency).^7^, ^8^, ^9^ These assays are also reported to detect antibodies for a more extended period compared with other assays.^10^

### 2.3 Data analysis

The main endpoint was vaccine‐induced and infection‐induced seroprevalence. For the main estimates as well as prefecture‐ and sex‐specific estimates, we further calculated estimates weighted by age group. We also conducted logistic regression to identify associations between various factors and past infection, adjusting for age group, sex, city of residence, and/or vaccination status. These potential confounders were determined a priori. We used Kruskal–Wallis test with Dunn's test to compare anti‐S antibody titers. Data analyses were performed using STATA version 17.0, GraphPad Prism 9.3.1, and R 4.1.2.

## 3 RESULTS

### 3.1 Characteristics of the study participants

Among 75 000 individuals invited, 16 296 agreed to participate: 8147 in the survey in December 2021 and 8149 in the survey in February–March 2022 (response rate 21.7%). Their attributes are shown in Table 1. The median age was 53 years (interquartile range [IQR] 43–64) with more females (9512; 58.4%) than males (6777; 41.6%). Compared with the general population of Japan, a smaller proportion of individuals were in their 20s (6.6% vs. 12.0% in the study and general population, respectively) and ≥70 years (16.3% vs. 27.1%), whereas more individuals were between their 40s and 60s (63.9% vs. 47.8%).^11^ The proportion of individuals who had received at least one dose was 96.2%, which was higher than the vaccination coverage among individuals ≥20 years in Japan (87.5% as of December 1, 2021, and 88.0% as of February 1, 2022). The proportion of individuals with self‐reported past SARS‐CoV‐2 infection was similar to the cumulative number of reported cases per 100 population in Japan when stratified by prefecture and age group, with only a few exceptions, including the 20s group in Tokyo in the December 2021 survey (Table S1). Participants' characteristics were similar between the December 2021 survey and February–March 2022 survey, except that higher proportions of individuals were vaccinated and/or infected in the latter (Table 1).

**TABLE 1:** Demographic and clinical characteristics of the study participants

	December 2021 survey (*n* = 8147) *n* (%)	February 2022 survey (*n* = 8149) *n* (%)	Total (*n* = 16 296) *n* (%)
Prefecture			
Miyagi	1700 (20.9)	1814 (22.3)	3514 (21.6)
Tokyo	2036 (25.0)	1912 (23.5)	3948 (24.2)
Aichi	1581 (19.4)	1521 (18.7)	3102 (19.0)
Osaka	1455 (17.9)	1353 (16.6)	2808 (17.2)
Fukuoka	1375 (16.9)	1549 (19.0)	2924 (17.9)
Sex			
Male	3334 (40.9)	3443 (42.3)	6777 (41.6)
Female	4810 (59.1)	4702 (57.7)	9512 (58.4)
Other/missing	3	4	7
Age in years			
20–29	521 (6.4)	560 (6.9)	1081 (6.6)
30–39	1046 (12.8)	1087 (13.3)	2133 (13.1)
40–49	1834 (22.5)	1751 (21.5)	3585 (22.0)
50–59	1919 (23.6)	1917 (23.5)	3836 (23.6)
60–69	1479 (18.2)	1516 (18.6)	2995 (18.4)
70–79	1041 (12.8)	1030 (12.6)	2071 (12.7)
80–89	289 (3.6)	271 (3.3)	560 (3.4)
90–99	15 (0.2)	14 (0.2)	29 (0.2)
Missing	3	3	6
Vaccination status			
None	316 (3.9)	296 (3.6)	612 (3.8)
Once	23 (0.3)	14 (0.2)	37 (0.2)
Twice	7759 (95.3)	6075 (74.6)	13 834 (84.9)
Three times	48 (0.6)	1762 (21.6)	1810 (11.1)
Missing	1	2	3
Past SARS‐CoV‐2 infection			
Yes	132 (1.6)	230 (2.8)	362 (97.8)
No	8014 (98.4)	7918 (97.2)	15 932 (2.2)
Missing	1	1	2
Occupation/industry			
Unemployed	1010 (12.4)	989 (12.1)	1999 (12.3)
Desk work	1243 (15.3)	1253 (15.4)	2496 (15.3)
Service (other than food and drinks)	722 (8.9)	736 (9.0)	1458 (9.0)
Healthcare	636 (7.8)	572 (7.0)	1208 (7.4)
Long‐term care	229 (2.8)	239 (2.9)	468 (2.9)
Manufacturing	509 (6.3)	532 (6.5)	1041 (6.4)
Management	445 (5.6)	448 (5.5)	903 (5.5)
Engineering/research	429 (5.3)	444 (5.5)	873 (5.4)
Self‐employed	317 (3.9)	371 (4.6)	688 (4.2)
Service (food and drinks)	213 (2.6)	210 (2.6)	423 (2.6)
Education	202 (2.5)	187 (2.3)	389 (2.4)
Transport	146 (1.8)	166 (2.0)	312 (1.9)
Childcare	125 (1.5)	139 (1.7)	264 (1.6)
Agriculture, forestry, and fisheries	63 (0.8)	80 (1.0)	143 (0.9)
Student	66 (0.8)	65 (0.8)	131 (0.8)
Housewives/househusband	1187 (14.6)	1060 (13.0)	2247 (13.8)
Multiple	65 (0.8)	108 (1.3)	173 (1.1)
Others	525 (6.5)	547 (6.7)	1072 (6.6)
Missing	5	3	8
Comorbidity			
Hypertension	1532 (19.3)	1561 (19.7)	3093 (19.5)
Heart disease	315 (4.0)	269 (3.4)	584 (3.7)
Diabetes mellitus	476 (6.0)	501 (6.3)	977 (6.2)
Kidney disease	77 (1.0)	78 (1.0)	155 (1.0)
Asthma	213 (2.7)	197 (2.5)	410 (2.6)
Chronic obstructive pulmonary disease	25 (0.3)	21 (0.3)	46 (0.3)
Obesity	260 (3.3)	299 (3.8)	559 (3.5)
Cancer	261 (3.3)	242 (3.1)	503 (3.2)
Immunodeficiency	6 (0.1)	7 (0.1)	13 (0.1)
Immunosuppressant use	54 (0.7)	38 (0.5)	92 (0.6)
No comorbidities	2404 (30.3)	2390 (30.1)	4794 (30.2)
Missing	224	210	434

### 3.2 Presence of SARS‐CoV‐2 antibodies: Overall and by age, sex, and prefecture

**FIGURE 2:**
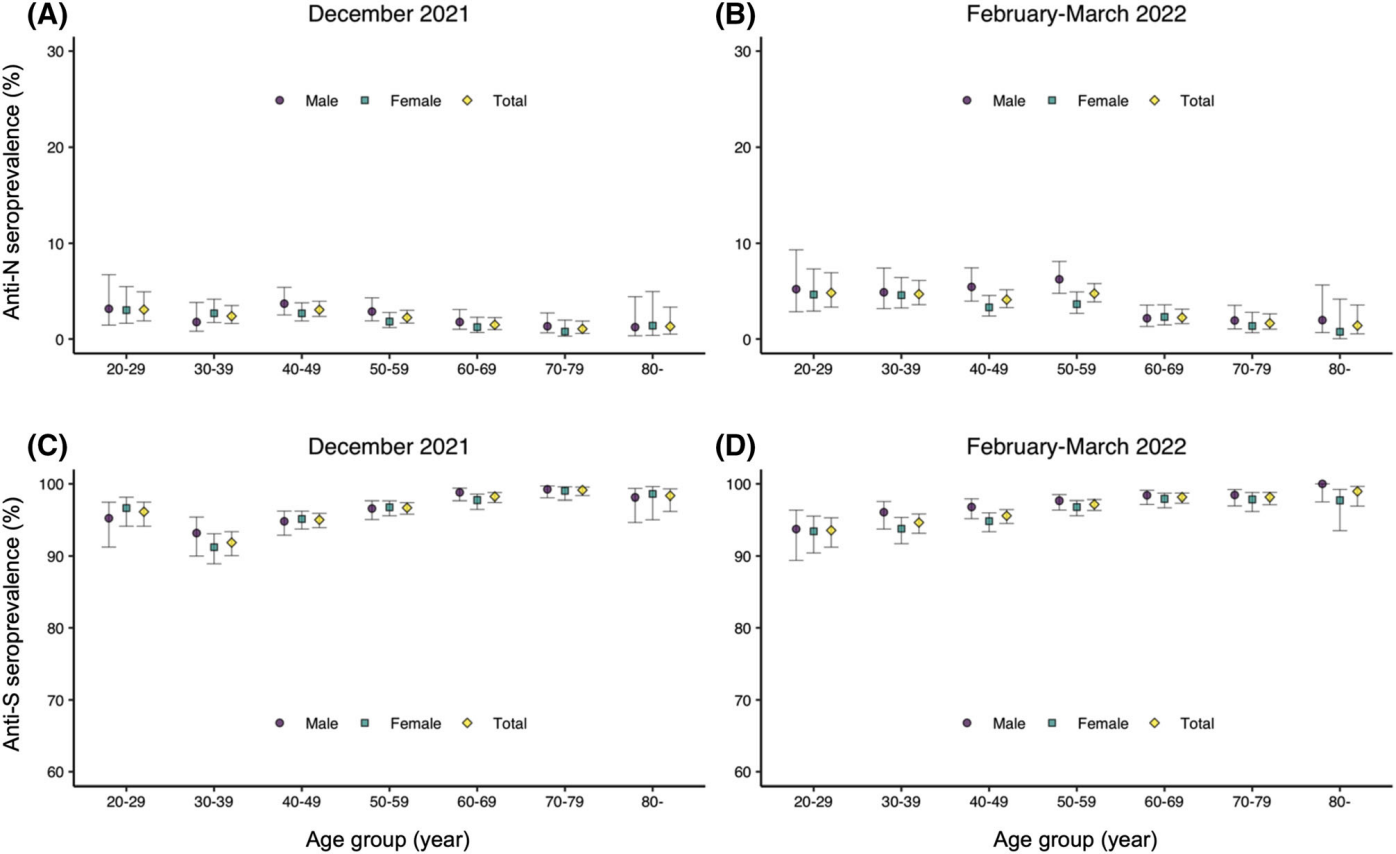
Proportions of individuals with infection‐induced and vaccine/infection‐induced antibodies by age and sex. (A) Proportion of individuals with anti‐N antibodies (infection‐induced antibodies) in the December 2021 survey. (B) Proportion of individuals with anti‐S antibodies (infection/vaccination‐induced antibodies) in the December 2021 survey. (C) Proportion of individuals with anti‐N antibodies in the February–March 2022 survey. (D) Proportion of individuals with anti‐S antibodies in the February–March 2022 survey. Error bars indicate 95% confidence intervals. Anti‐N: anti‐nucleocapsid; anti‐S: anti‐spike antibodies

Among all participants, after weighting for age groups, the overall prevalence of infection‐induced antibodies (anti‐N antibodies) was 2.2% (95% confidence interval [95% CI], 1.9%–2.5%) in the December 2021 survey and 3.5% (95% CI, 3.1%–3.9%) in the February–March 2022 survey (Table S2). The overall prevalence of vaccine/infection‐induced antibodies (anti‐S antibodies) was 96.3% (95% CI, 95.8%–96.6%) in the December 2021 survey and 96.5% (95% CI, 96.1%–96.9%) in the February–March 2022 survey. The proportions of individuals with infection‐induced and vaccine/infection‐induced antibodies by age group and sex or prefecture are illustrated in Figures 2 and S2. Age‐ and prefecture‐specific infection‐induced seroprevalence ranged from 0.0% (≥70 in Aichi and 60s in Fukuoka) to 7.0% (20s in Fukuoka) in the December 2021 survey and from 0.0% (80s in both Miyagi and Fukuoka) to 8.4% (20s in Osaka) in the February–March 2022 survey. Vaccine/infection‐induced seroprevalence ranged from 83.6% (30s in Osaka) to 100.0% (80s in Fukuoka) in the December 2021 survey and from 89.4% (20s in Fukuoka) to 100.0% (≥80 in Aichi and Fukuoka) in the February–March 2022 survey. The seroprevalence was generally similar between males and females for both infection‐induced and vaccine/infection‐induced antibodies, except that the infection‐induced seroprevalence was higher for males among the 40–50s in the February–March 2022 survey (Figure 2).

### 3.3 Past SARS‐CoV‐2 infection and presence of the infection‐induced antibodies

The proportion of individuals with a self‐reported past diagnosis history of SARS‐CoV‐2 infection among those who had infection‐induced antibodies was 59% (105/177; 95% CI, 52%–67%) during the December 2021 survey and 60% (178/296; 95% CI, 54%–66%) during the February–March 2022 survey. The infection‐induced seroprevalence among individuals with a self‐reported past diagnosis history of infection was 80% (105/132; 95% CI, 71%–86%) during the December 2021 survey and 77% (178/230; 95% CI, 71%–83%) during the February–March 2022 survey without apparent waning over time (Table S3).

### 3.4 Sociodemographic and other factors associated with past SARS‐CoV‐2 infection

We next described the proportion of individuals with past SARS‐CoV‐2 infection stratified by various factors and further analyzed the association between these factors and past infection. Based on the above findings of partial overlap between self‐reported history of SARS‐CoV‐2 infection and infection‐induced antibodies, we performed these analyses with past infection defined as either the presence of anti‐N antibodies or self‐reported history of past SARS‐CoV‐2 infection. Overall, 2.5% (95% CI, 2.2%–2.9%) and 4.3% (95% CI, 3.8%–4.7%) of participants had a past infection during the December 2021 survey and the February–March 2022 survey, respectively (Table 2). The odds of infection were two to three times higher among individuals in urban prefectures (Tokyo and Osaka) than the rural prefecture (Miyagi). The odds of infection were slightly lower in females compared with males. These trends were more evident in the February–March 2022 survey than in the December 2021 survey. Compared with individuals in 20s, individuals in ≥60s had approximately half the odds of infection in both surveys. An increase in the proportion of individuals with past infection was observed for almost all age groups. After adjusting for age group, sex, and city of residence, individuals vaccinated at least twice had lower odds of infection compared with unvaccinated individuals. Several types of occupations were associated with higher odds of infection compared with unemployment: Education (adjusted odds ratio [aOR] 2.91; 95% CI, 1.28–6.62) and long‐term care workers (aOR 3.13; 95% CI, 1.47–6.66) in the December 2021 survey and management positions (aOR 2.38; 95% CI, 1.27–4.45), service industry for food and drinks (aOR 3.09; 95% CI, 1.50–6.35), and childcare (aOR 3.63; 95% CI, 1.60–8.24) in the February–March 2022 survey. Approximately 45% of individuals who had household contact with infected individuals had evidence of past infection, resulting in much higher odds of infection compared with those who did not have contact. Also, 11.8% and 18.7% of individuals, respectively, who had non‐household contact with infected individuals had evidence of past infection during the December 2021 survey and the February–March 2022 survey, respectively, resulting in higher odds of infection.

**TABLE 2:** Proportion of individuals with past infection and odds ratio of past infection by prefecture, vaccination status, occupation, and close contact history

	December 2021 survey (*n* = 8147)		February 2022 survey (*n* = 8149)	
	*n* (%) (95% CI)	Odds ratio (crude or adjusted)	*n* (%) (95% CI)	Odds ratio (crude or adjusted)
All	2.5 (2.2–2.9)	N/A	4.3 (3.8–4.7)	N/A
Prefecture^a^				
Miyagi	1.5 (1.0–2.2)	1	2.0 (1.4–2.7)	1
Tokyo	3.1 (2.5–4.0)	2.17 (1.36–3.47)	6.4 (5.3–7.6)	3.37 (2.31–4.91)
Aichi	1.8 (1.3–2.6)	1.25 (0.73–2.15)	3.7 (2.8–4.8)	1.89 (1.24–2.89)
Osaka	4.1 (3.2–5.3)	2.88 (1.80–4.62)	6.1 (5.0–7.5)	3.23 (2.17–4.80)
Fukuoka	1.9 (1.3–2.8)	1.29 (0.74–2.25)	3.3 (2.5–4.3)	1.68 (1.09–2.59)
Sex^a^				
Male	2.8 (2.3–3.4)	1	4.9 (4.2–5.6)	1
Female	2.3 (1.9–2.8)	0.84 (0.64–1.11)	3.8 (3.3–4.4)	0.79 (0.63–0.97)
Age in years^a^				
20–29	4.0 (2.7–6.1)	1	5.9 (4.2–8.2)	1
30–39	2.5 (1.7–3.6)	0.61 (0.34–1.09)	5.3 (4.2–6.8)	0.90 (0.58–1.40)
40–49	3.2 (2.5–4.1)	0.78 (0.47–1.29)	4.5 (3.6–5.6)	0.75 (0.50–1.15)
50–59	2.5 (1.9–3.3)	0.61 (0.36–1.03)	5.2 (4.3–6.3)	0.88 (0.59–1.32)
60–69	1.8 (1.3–2.6)	0.44 (0.25–0.79)	2.8 (2.1–3.8)	0.47 (0.29–0.74)
70–79	1.6 (1.0–2.6)	0.40 (0.21–0.76)	2.5 (1.7–3.7)	0.41 (0.24–0.70)
80–89	2.1 (1.0–4.5)	0.50 (0.20–1.27)	3.3 (1.8–6.2)	0.55 (0.26–1.16)
90–99	6.7 (1.2–29.8)	1.70 (0.21–13.5)	0 (0–21.5)	N/A
Vaccination status^b^				
None	7.6 (5.2–11.1)	1	10.5 (7.5–14.5)	1
Once	8.7 (2.4–26.8)	1.23 (0.27–5.73)	28.6 (11.7–54.6)	3.45 (0.98–12.2)
Twice	2.3 (1.9–2.6)	0.32 (0.20–0.51)	4.6 (4.1–5.1)	0.41 (0.28–0.61)
Three times	6.3 (2.1–16.9)	0.79 (0.22–2.78)	1.9 (1.4–2.7)	0.21 (0.12–0.36)
Fields/types of occupation^c^				
Unemployed	1.8 (1.1–2.8)	1	2.1 (1.4–3.2)	1
Desk work	2.4 (1.7–3.4)	1.07 (0.53–2.13)	4.3 (3.3–5.6)	1.57 (0.88–2.82)
Service (other than food and drinks)	2.9 (1.9–4.4)	1.28 (0.62–2.64)	4.1 (2.9–5.8)	1.45 (0.77–2.72)
Healthcare workers	2.2 (1.3–3.7)	0.99 (0.44–2.27)	2.8 (1.7–4.5)	1.77 (0.84–3.74)
Long‐term care facility workers	2.2 (0.9–5.0)	1.07 (0.37–3.10)	5.9 (3.5–9.6)	3.13 (1.47–6.66)
Manufacturing	1.6 (0.8–3.1)	0.71 (0.28–1.75)	4.1 (2.7–6.2)	1.45 (0.74–2.82)
Management position	3.3 (2.0–5.4)	1.30 (0.60–2.82)	7.1 (5.1–9.9)	2.38 (1.27–4.45)
Engineering/research	2.3 (1.3–4.2)	0.90 (0.38–2.12)	4.7 (3.1–7.1)	1.38 (0.70–2.71)
Self‐employed	3.2 (1.7–5.7)	1.39 (0.60–3.21)	5.1 (3.3–7.9)	1.86 (0.95–3.64)
Service (food and drinks)	4.7 (2.6–8.4)	2.01 (0.84–4.83)	8.1 (5.1–12.6)	3.09 (1.50–6.35)
Education	5.9 (3.4–10.1)	2.91 (1.28–6.62)	3.2 (1.5–6.8)	1.19 (0.45–3.15)
Transport	1.4 (3.8–4.9)	0.57 (0.12–2.57)	3.0 (1.3–6.9)	1.05 (0.37–2.92)
Childcare	0.8 (0.1–4.4)	0.45 (0.06–3.52)	7.9 (4.5–13.6)	3.63(1.60–8.24)
Agriculture, forestry, and fisheries	1.6 (0.3–8.5)	1.37 (0.17–10.8)	2.5 (0.7–8.7)	1.58 (0.35–7.09)
Student	3.0 (0.8–10.4)	0.88 (0.18–4.30)	6.2 (2.4–14.8)	1.59 (0.48–5.26)
Housewives/househusband	2.4 (1.6–3.4)	1.40 (0.72–2.74)	3.1 (2.2–4.3)	1.55 (0.85–2.83)
Multiple	6.2 (2.4–14.8)	2.70 (0.83–8.80)	4.6 (2.0–10.4)	1.51 (0.53–4.31)
Others	2.5 (1.5–4.2)	1.20 (0.55–2.61)	6.6 (4.8–9.0)	2.67 (1.48–4.83)
Household contact^c^				
No	1.6 (1.4–1.9)	1	2.6 (2.3–3.0)	1
Yes	44.4 (37.0–52.1)	48.4 (32.9–71.4)	44.8 (39.4–50.3)	26.4 (20.0–34.8)
Non‐household contact^c^				
No	2.1 (1.8–2.5)	1	3.7 (3.3–4.1)	1
Yes	11.8 (8.5–16.1)	5.16 (3.42–7.77)	18.7 (14.8–23.2)	5.21 (3.80–7.14)

*Note*: Past infection defined by the presence of anti‐nucleocapsid (N) antibodies and/or history of past PCR/antigen‐positive.

^a^ Crude odds ratio.

^b^ Adjusted for age group, sex, and city of residence.

^c^ Adjusted for age group, sex, vaccination status, and city of residence.

### 3.5 Description of vaccinated individuals who did not develop detectable antibodies

Among 15 681 individuals who reported to have been vaccinated at least once, only 11 individuals (0.07%) did not develop detectable anti‐S antibodies. Of these 11 individuals, eight were ≥60s, seven had cancer, and two used immunosuppressants.

### 3.6 Quantitative assessment of anti‐S antibody titers

**FIGURE 3:**
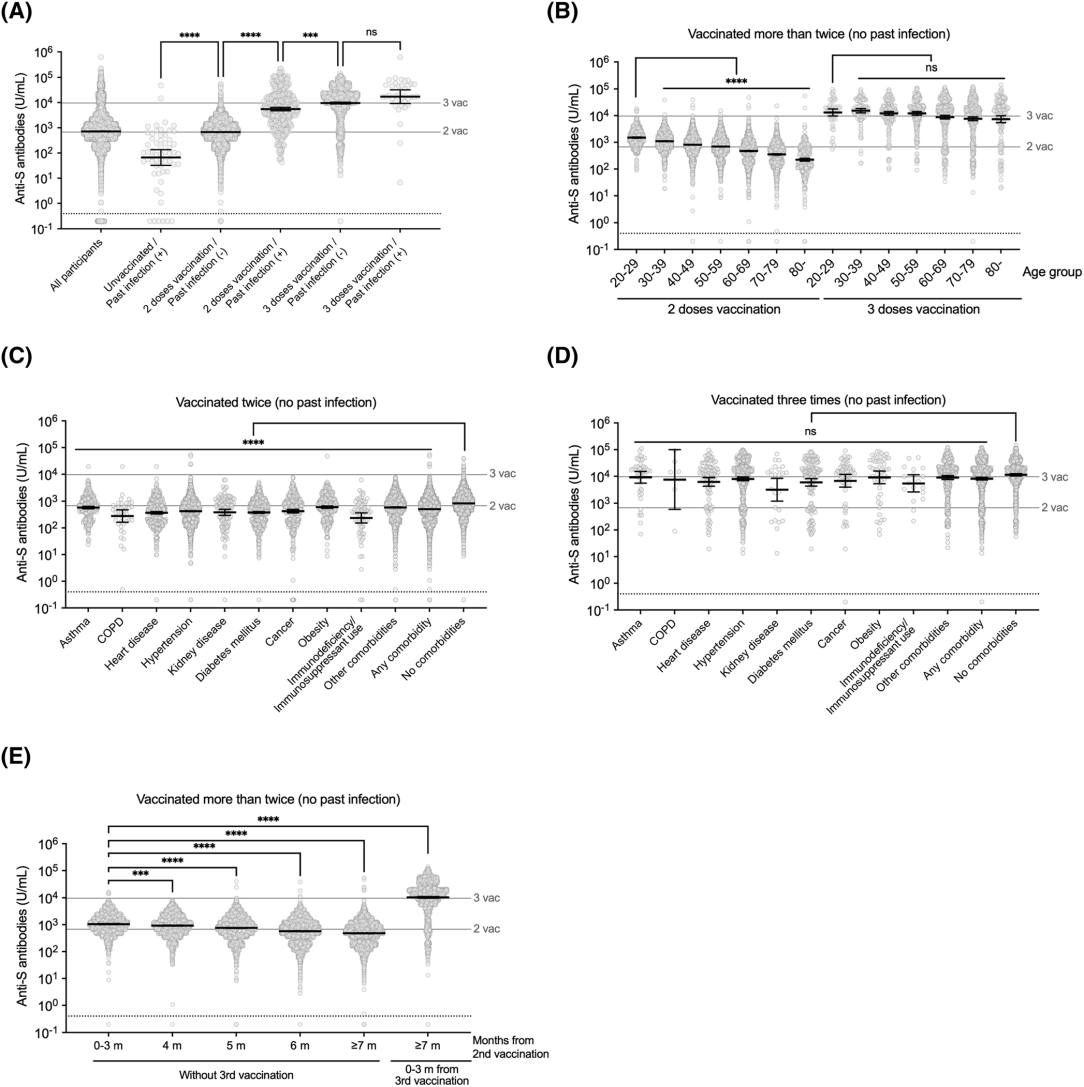
Quantitative assessment of anti‐S antibody titers. (A) Anti‐S antibody (infection/vaccination‐induced antibody) titers by COVID‐19 vaccination status and past SARS‐CoV‐2 infection history determined by self‐reported history of past infection or presence of the infection‐induced antibodies. The titers between groups with different vaccination status and past infection history were compared using Kruskal–Wallis test with Dunn's test. (B) Anti‐S antibody titers by vaccination status and age group. The titers between 20–29 age group and older age groups with same vaccination status were compared using Kruskal–Wallis test with Dunn's test. (C) Anti‐S antibody titers by underlying disease among individuals who received two doses of COVID‐19 vaccines without evidence of past infection. COPD; chronic obstructive pulmonary disease. The titers between those with and without comorbidities were compared using Kruskal–Wallis test with Dunn's test. (D) Anti‐S antibody titers by underlying disease among individuals who received three doses of COVID‐19 vaccines without evidence of past infection. The titers between those with and without comorbidities were compared using Kruskal–Wallis test with Dunn's test; ns, not significant. (E) Anti‐S antibody titers by time since the second dose. The titers between those who were after 0–3 months since second dose and those with longer time than 4 months since the second dose or those with the third dose were compared using Kruskal–Wallis test with Dunn's test. A black bar on each plot indicates geometric mean with 95% confidence interval. Anti‐S antibody; anti‐spike antibodies. The horizontal gray bar line with “3 vac” or “2vac” label indicates geometric mean titer of anti‐S antibody titers of individuals who received three doses or two doses of COVID‐19 vaccines without evidence of past infection, respectively. The horizontal dotted line without any label indicates manufacturer‐determined cutoff. For (C), and (D), groups are not mutually exclusive. Statistical significance: ns, not significant, ***p < 0.001, ****p < 0.0001

We quantitatively assessed the levels of anti‐S antibodies by number of vaccine doses received and presence of SARS‐CoV‐2 infection history (a self‐reported history of past SARS‐CoV‐2 infection or presence of the infection‐induced antibodies). Individuals who received two doses of vaccine without evidence of past infection had higher levels of antibodies than previously infected unvaccinated individuals (Figure 3A). Individuals who received two doses with a previous infection had higher levels of antibodies than those who received two doses of vaccine without evidence of past infection. In addition, individuals who received three doses of vaccine with or without evidence of past infection had similar antibody titers at a level higher than individuals, who received two doses of vaccine with a previous infection. We next looked at levels of antibodies by age group and the number of vaccine doses received (Figure 3B). There was a decreasing trend in antibody titers as the age increased. Individuals who received three doses of vaccines had higher titers than those who received two, regardless of age group. When we examined anti‐S antibody titers among vaccinees with underlying diseases, individuals who received two doses of vaccine with an underlying disease had lower anti‐S antibodies than those without (Figure 3C). This was especially evident among individuals with heart disease, diabetes mellitus, cancer, and immunodeficiency, or who used immunosuppressants. In contrast, individuals who received three doses of vaccine with an underlying disease had similar antibody titers to those without (Figure 3D). Finally, we examined antibody titers by time since the second vaccination (Figure 3E). We observed a slight decrease in antibody titers over time but a robust increase after the third dose.

## 4 DISCUSSION

In this national seroepidemiological investigation conducted before and during the peak of the Omicron‐dominant period in Japan with over 15 000 individuals, we examined the cumulative burden of SARS‐CoV‐2 infection and assessed the extent of antibody development among the COVID‐19 vaccinees.

The proportion of individuals with infection‐induced antibodies was only 2.2% before the Omicron wave and 3.6% around the peak of the Omicron wave. Although younger people and people living in urban areas had higher seroprevalence than older people and people living in rural areas, the infection‐induced seroprevalence remained <10% even among the 20s in Tokyo and Osaka, where the burden of COVID‐19 has been the highest throughout the pandemic. This was in stark contrast to other countries, where seroprevalence studies were conducted during the same periods, using the same commercial assays.^6^, ^12^, ^13^ In the United States, infection‐induced seroprevalence rose from 33.5% in December 2021 to 57.7% in February 2022, reaching 63.7% among individuals aged 18–49 years.^12^ Similarly, in the United Kingdom, infection‐induced seroprevalence was approximately 25% in December 2021 and 45% in March 2022, with a prevalence of around 60% among individuals aged 17–29 years.^13^ Furthermore, a systematic review and meta‐analysis conducted by the WHO estimated the global seroprevalence to be 45.2% (95% CI, 40.7%–49.8%) in July 2021.^6^ Our findings and those of other studies in the United States, the United Kingdom, and globally indicate that the cumulative COVID‐19 burden has been over 10 times lower in Japan. Many factors may have contributed to this, such as extensive contact tracing by public health centers, including backward tracing (source investigation), high mask‐wearing adherence, maintaining greater physical distance, appropriate targeting of the policies/risk communication, and strict infection prevention and control measures at healthcare/long‐term care facilities.^14^, ^15^ The contribution of each factor remains unknown.

The proportion of individuals with a self‐reported history of past SARS‐CoV‐2 infection among individuals who had infection‐induced antibodies was approximately 60% in both surveys, equaling seroprevalence to confirmed case ratios of 1.67. This number was comparable to what was observed in high‐income countries in the Americas and Europe and much lower than those in other countries, refuting the criticism that Japan was not testing enough, resulting in lower reported case counts.^16^ Interestingly, approximately 80% of participants with a self‐reported history of past SARS‐CoV‐2 infection did not have detectable infection‐induced seroprevalence, which is lower than expected from the assay sensitivity. This could be because >90% of individuals were vaccinated in this study population and past reports suggest that vaccinated individuals develop anti‐N antibodies to a lesser extent.^17^

Although the overall burden of infection was low in Japan, some factors were associated with higher odds of infection, such as residing in urban prefectures, younger age, and not being vaccinated. Careful interpretation is necessary as individuals vaccinated could have been infected before vaccination; thus, lower odds among vaccinees are not entirely a causal effect of the vaccination. Some occupations were associated with higher odds of infection. Notably, long‐term care workers, having higher odds of infection, suggest that they could have passed SARS‐CoV‐2 down to elderlies who have higher risks of severe COVID‐19. Increased odds of infection among childcare workers were observed in the survey conducted during the peak of the Omicron wave, which could indirectly reflect a higher burden of the Omicron variant on children compared with that of previous variants as observed through case notifications.^18^, ^19^ Food service workers had higher odds of infection, possibly because customers at bars and restaurants had opportunities to take their masks off while dining.^14^ Almost half (45%) of the individuals who had household contact and 10–20% with non‐household contact were infected; these numbers may reflect accurate secondary attack rates through incorporation of serology data and were similar or higher compared with those in other reports.^20^

We finally observed that almost all vaccinated individuals developed detectable antibodies except for very few of those who were mostly elderly or immunosuppressed. Quantitative assessment of antibodies revealed that, although there was a decreasing trend in antibody titers as the age increased, and individuals with underlying diseases tended to have lower anti‐S antibodies partly owing to the rollout timing, individuals who received three doses of vaccines had higher titers than individuals who received two doses, regardless of the age group. Although correlates of protection for infection, symptom development, and severe disease are not well understood,^21^, ^22^, ^23^ these results were reassuring and, together with published epidemiological evidence,^24^, ^25^, ^26^ support a continued rollout of a third booster dose.

### 4.1 Limitations

First, the low response rate may have resulted in selection bias. There was a slight difference in the distribution of age groups compared with the background population, but seroprevalence calculation stratified by age groups at least partially overcame this difference. Also, because the proportion of individuals with self‐reported past SARS‐CoV‐2 infection in this study was similar to the cumulative number of reported COVID‐19 cases per 100 population in Japan when stratified by prefecture and age group, we assumed that the participants are similar to the population in terms of risk of infection (Table S1). Because the vaccination rate was higher among the study population than among the baseline population, the infection‐induced seroprevalence may be underestimated and anti‐S seroprevalence may be overestimated. Second, we used self‐reported questionnaires to obtain metadata, so biases such as recall bias and social desirability bias are possible. Third, we focused on five prefectures for this investigation, with four in urban prefectures; hence, the actual prevalence in the entire nation may be even lower. Fourth, we did not include individuals <20 years of age. Fifth, the serology assays used have high sensitivity/specificity, but the waning of antibodies may lead to the misclassification of serostatus. However, this concern may be minor in Japan, where the infection burden was very low before the Delta wave (Figure 1).

### 4.2 Conclusions

This national seroepidemiological investigation indicates that the overall burden of infection was much lower in Japan than in other countries. The majority of the population remained infection‐naïve at the peak of the Omicron‐dominant period but developed anti‐SARS‐CoV‐2 spike antibodies owing to vaccination. Despite the low overall burden, some sociodemographic groups such as those residing in urban prefectures, younger age groups, being unvaccinated, and individuals engaged in occupations such as long‐term care workers, childcare workers, and food service workers had higher odds of past infection.

Our study complemented and supported the routine surveillance activity to provide a better picture of the cumulative burden of COVID‐19.

## CONFLICT OF INTEREST

The authors declare no conflicts of interest.

## ETHICS STATEMENT

The ethics committee of the National Institute of Infectious Diseases approved our study (NIID 1312). Written informed consent was obtained from all participants.

## AUTHOR CONTRIBUTIONS

**Takeshi Arashiro:** Conceptualization; data curation; formal analysis; investigation; methodology; validation; visualization; writing‐original draft; writing‐review and editing. **Satoru Arai:** Conceptualization; investigation; project administration; writing‐review and editing. **Ryo Kinoshita:** Data curation; formal analysis; validation; visualization; writing‐review and editing. **Kanako Otani:** Data curation; formal analysis; validation; writing‐review and editing. **Sho Miyamoto:** Data curation; formal analysis; validation; visualization; writing‐review and editing. **Daisuke Yoneoka:** Data curation; formal analysis; validation; writing‐review and editing. **Taro Kamigaki:** Data curation; formal analysis; validation; visualization; writing‐review and editing. **Hiromizu Takahashi:** Conceptualization; funding acquisition; investigation; writing‐review and editing. **Hiromi Hibino:** Conceptualization; funding acquisition; investigation; writing‐review and editing. **Mai Okuyama:** Investigation. **Ai Hayashi:** Investigation. **Fuka Kikuchi:** Investigation. **Saeko Morino:** Investigation; writing‐review and editing. **Sayaka Takanashi:** Investigation. **Takaji Wakita:** Funding acquisition; investigation; resources; writing‐review and editing. **Keiko Taya:** Conceptualization; investigation; writing‐review and editing. **Tadaki Suzuki:** Conceptualization; data curation; formal analysis; funding acquisition; investigation; project administration; supervision; validation; visualization; writing‐original draft; writing‐review and editing. **Motoi Suzuki:** Conceptualization; funding acquisition; investigation; supervision; writing‐review and editing.

### PEER REVIEW

The peer review history for this article is available at https://publons.com/publon/10.1111/irv.13094.

## Supporting information


**Table S1.** Proportion of population with individuals who reported to have past SARS‐CoV‐2 infection in the study population and cumulative proportion of reported COVID‐19 cases per 100 population in Japan by prefecture and age group
**Table S2.** Crude seroprevalence and seroprevalence weighted by age group
**Table S3.** The infection‐induced seroprevalence among individuals with a past diagnosis history of SARS‐CoV‐2 infection
**Figure S1.** Sampling frame.
**Figure S2.** Proportions of individuals with infection‐induced and vaccine/infection‐induced antibodies by age and prefecture. (A) Proportion of individuals with anti‐N antibodies (infection‐induced antibodies) in the December 2021 survey. (B) Proportion of individuals with anti‐S antibodies (infection/vaccination‐induced antibodies) in the December 2021 survey. (C) Proportion of individuals with anti‐N antibodies in the February–March 2022 survey. (D) Proportion of individuals with anti‐S antibodies in the February–March 2022 survey. Error bars indicate 95% confidence intervals. Anti‐N: anti‐nucleocapsid; anti‐S: anti‐spike antibodies.

## Data Availability

Individual‐level data of patients included in this manuscript after de‐identification are considered sensitive and will not be shared. The study methods and statistical analyses are all described in detail in Section 2 and throughout the manuscript.
