# Supplementary material for: National seroepidemiological study of COVID‐19 after the initial rollout of vaccines: Before and at the peak of the Omicron‐dominant period in Japan
Source: Influenza Other Respir Viruses. 2023 Feb 1;17(2):e13094. doi: 10.1111/irv.13094 (PMC9890143; doi:10.1111/irv.13094)

**Supporting Information**

**Table S1.** Proportion of population with individuals who reported to have past SARS-CoV-2 infection in the study population and cumulative proportion of reported COVID-19 cases per 100 population in Japan by prefecture and age group

|  | **December 2021 survey** | | **February-March 2022 survey** | |
| --- | --- | --- | --- | --- |
|  | **Survey participants**  % (95% CI) | **Reported cases in Japan (by the end of November 2021)**  % | **Survey participants**  % (95% CI) | **Reported cases in Japan (by the end of January 2022)**  % |
| **Miyagi (age in years)** |  |  |  |  |
| 20-29 | 1.5 (0.9-7.6) | 1.67 | 1.7 (0.5-5.9) | 2.12 |
| 30-39 | 0.4 (0.1-2.6) | 1.02 | 2.0 (0.9-4.7) | 1.30 |
| 40-49 | 1.0 (0.0-2.6) | 0.75 | 1.2 (0.5-2.7) | 0.93 |
| 50-59 | 0.8 (0.3-2.3) | 0.62 | 1.3 (0.5-3.0) | 0.73 |
| 60-69 | 0.9 (0.3-2.6) | 0.38 | 0.9 (0.3-2.5) | 0.45 |
| 70-79 | 0.0 (0-1.7) | 0.30 | 1.3 (0.4-3.8) | 0.34 |
| 80+ | 1.9 (0.3-9.7) | 0.38 | 3.1 (0.8-10.5) | 0.42 |
| Total | 0.9 (0.5-1.5) | 0.71 | 1.4 (0.9-2.0) | 0.92 |
| **Tokyo (age in years)** |  |  |  |  |
| 20-29 | 0.0 (0.0-2.9) | 5.98 | 5.2 (2.7-10.0) | 9.12 |
| 30-39 | 1.7 (0.7-4.0) | 3.92 | 7.3 (4.8-10.7) | 6.03 |
| 40-49 | 3.2 (2.0­-5.3) | 2.74 | 5.2 (3.4-7.7) | 4.28 |
| 50-59 | 1.8 (1.0-3.4) | 2.25 | 5.9 (4.1-8.5) | 3.30 |
| 60-69 | 2.1 (1.0-4.3) | 1.36 | 1.6 (0.7-3.6) | 2.07 |
| 70-79 | 1.9 (0.8-4.4) | 0.92 | 1.9 (0.7-4.7) | 1.35 |
| 80+ | 3.0 (0.8-10.5) | 1.16 | 5.3 (1.8-14.6) | 1.64 |
| Total | 2.1 (1.6-2.8) | 2.72 | 4.7 (3.8-5.8) | 4.34 |
| **Aichi (age in years)** |  |  |  |  |
| 20-29 | 1.6 (0.4-5.7) | 3.32 | 4.2 (1.8-9.4) | 5.29 |
| 30-39 | 0.5 (0.08-2.6) | 1.93 | 2.9 (1.3-6.2) | 3.26 |
| 40-49 | 2.1 (1.0-4.4) | 1.45 | 2.1 (1.0-4.3) | 2.40 |
| 50-59 | 1.5 (0.7-3.2) | 1.17 | 3.4 (2.0-5.9) | 1.85 |
| 60-69 | 0.0 (0.0-1.3) | 0.74 | 0.7 (0.2-2.6) | 1.19 |
| 70-79 | 1.1 (0.3-4.0) | 0.54 | 0.6 (0.1-3.0) | 0.84 |
| 80+ | 1.8 (0.3-9.6) | 0.75 | 1.8 (0.3-9.3) | 1.12 |
| Total | 1.2 (0.8-1.9) | 1.42 | 2.2 (1.6-3.1) | 2.43 |
| **Osaka (age in years)** |  |  |  |  |
| 20-29 | 3.5 (1.2-9.8) | 5.02 | 7.2 (0.3-14.9) | 8.36 |
| 30-39 | 1.7 (0.6-4.9) | 3.14 | 4.8 (2.3-9.6) | 5.31 |
| 40-49 | 3.6 (2.1-6.1) | 2.36 | 2.2 (1.0-4.6) | 3.91 |
| 50-59 | 3.5 (2.0-6.0) | 1.98 | 2.5 (1.3-4.8) | 3.06 |
| 60-69 | 1.6 (0.6-4.0) | 1.30 | 4.6 (2.6-7.8) | 1.99 |
| 70-79 | 1.2 (0.3-4.4) | 0.95 | 2.3 (0.9-5.7) | 1.38 |
| 80+ | 1.4 (0.2-7.3) | 1.28 | 1.8 (0.3-9.6) | 1.84 |
| Total | 2.6 (1.9-3.6) | 2.29 | 3.3 (2.5-4.4) | 3.92 |
| **Fukuoka (age in years)** |  |  |  |  |
| 20-29 | 7.0 (3.0-15.4) | 3.68 | 1.1 (0.2-6.4) | 5.88 |
| 30-39 | 1.9 (0.7-5.6) | 2.04 | 3.2 (1.5-6.8) | 3.27 |
| 40-49 | 0.7 (0.2-2.4) | 1.54 | 3.5 (1.9-6.3) | 2.40 |
| 50-59 | 1.3 (0.5-3.4) | 1.25 | 2.6 (1.4-4.8) | 1.80 |
| 60-69 | 0.4 (0.1-2.0) | 0.71 | 1.9 (0.9-4.0) | 1.02 |
| 70-79 | 0.9 (0.3-3.4) | 0.50 | 1.3 (0.4-3.7) | 0.69 |
| 80+ | 0 (0.0-6.4) | 0.67 | 0 (0-6.9) | 0.90 |
| Total | 1.2 (0.8-1.9) | 1.45 | 2.3 (1.7-3.2) | 2.34 |
| Total | 1.6 (1.4-1.9) | 2.07 | 2.8 (2.5-3.2) | 3.38 |

**Table S2.** Crude seroprevalence and seroprevalence weighted by age group

|  | **December 2021 survey** | | **February-March 2022 survey** | |
| --- | --- | --- | --- | --- |
|  | **Crude**  % (95% CI) | **Weighted by age group**  % (95% CI) | **Crude**  % (95% CI) | **Weighted by age group**  % (95% CI) |
| **Overall** |  |  |  |  |
| Anti-N antibodies^†^ | 2.2 (1.9-2.5) | 2.2 (1.9-2.5) | 3.6 (3.3-4.1) | 3.5 (3.1-3.9) |
| Anti-S antibodies^‡^ | 96.3 (95.9-96.7) | 96.3 (95.8-96.6) | 96.6 (96.2-97) | 96.5 (96.1-96.9) |
| **Prefecture: Miyagi** |  |  |  |  |
| Anti-N antibodies^†^ | 1.2 (0.8-1.8) | 1.2 (0.8-1.8) | 1.5 (1-2.2) | 1.4 (1-2.1) |
| Anti-S antibodies^‡^ | 97.2 (96.3-97.9) | 97.1 (96.2-97.8) | 96.9 (95.9-97.6) | 96.7 (95.8-97.5) |
| **Prefecture: Tokyo** |  |  |  |  |
| Anti-N antibodies^†^ | 2.8 (2.2-3.6) | 2.6 (2-3.3) | 5.7 (4.7-6.8) | 5.2 (4.3-6.3) |
| Anti-S antibodies^‡^ | 96.9 (96-97.5) | 97.2 (96.4-97.9) | 97.1 (96.3-97.8) | 97.3 (96.4-97.9) |
| **Prefecture: Aichi** |  |  |  |  |
| Anti-N antibodies^†^ | 1.6 (1.1-2.3) | 1.5 (1-2.2) | 3.1 (2.3-4.1) | 3.1 (2.3-4.1) |
| Anti-S antibodies^‡^ | 95.8 (94.7-96.7) | 96 (94.9-96.9) | 96.1 (95-97) | 96.4 (95.3-97.2) |
| **Prefecture: Osaka** |  |  |  |  |
| Anti-N antibodies^†^ | 3.8 (2.9-4.9) | 3.6 (2.8-4.7) | 5.3 (4.2-6.6) | 5 (4-6.3) |
| Anti-S antibodies^‡^ | 94.6 (93.4-95.7) | 94.2 (92.8-95.2) | 96.4 (95.2-97.2) | 95.8 (94.6-96.7) |
| **Prefecture: Fukuoka** |  |  |  |  |
| Anti-N antibodies^†^ | 1.5 (0.9-2.2) | 2.0 (1.4-2.9) | 2.7 (2-3.6) | 2.9 (2.2-3.9) |
| Anti-S antibodies^‡^ | 96.9 (95.8-97.7) | 96.2 (95.1-97.1) | 96.4 (95.4-97.3) | 95.9 (94.8-96.8) |
| **Sex: Male** |  |  |  |  |
| Anti-N antibodies^†^ | 2.4 (1.9-3) | 2.3 (1.9-2.9) | 4.2 (3.6-4.9) | 4.2 (3.5-4.9) |
| Anti-S antibodies^‡^ | 96.8 (96.1-97.3) | 96.4 (95.7-97) | 97.5 (96.9-97.9) | 97.1 (96.4-97.6) |
| **Sex: Female** |  |  |  |  |
| Anti-N antibodies^†^ | 2.0 (1.7-2.5) | 2.1 (1.7-2.5) | 3.2 (2.7-3.8) | 3 (2.6-3.6) |
| Anti-S antibodies^‡^ | 96.0 (95.4-96.5) | 96.2 (95.6-96.7) | 96 (95.4-96.5) | 96 (95.4-96.6) |

^†^anti-N antibodies: anti-nucleocapsid antibodies (infection-induced antibodies)

^‡^Anti-S antibodies: anti-spike antibodies (infection/vaccination-induced antibody)

**Table S3.** The infection-induced seroprevalence among individuals with a past diagnosis history of SARS-CoV-2 infection

|  | Time since diagnosis | | | Total |
| --- | --- | --- | --- | --- |
|  | **0-5 months** | **6-10 months** | **11 months-** |  |
| Past diagnosis history of infection  （Diagnosis month reported cases only） | 109 | 111 | 85 | 305 |
| Anti-N antibodies^†^ (+) | 95 | 106 | 77 | 278 |
| Seroprevalence %  (95% CI) | 87.2%  (79.6-92.2) | 95.5%  (90.0-98.1) | 90.6%  (82.5-95.2) | 91.1%  (87.4-93.8) |

^†^anti-N antibodies: anti-nucleocapsid antibodies (infection-induced antibodies)

**Figure S1.** Sampling frame.

**Figure S2.** Proportions of individuals with infection-induced and vaccine/infection-induced antibodies by age and prefecture. (A) Proportion of individuals with anti-N antibodies (infection-induced antibodies) in the December 2021 survey. (B) Proportion of individuals with anti-S antibodies (infection/vaccination-induced antibodies) in the December 2021 survey. (C) Proportion of individuals with anti-N antibodies in the February-March 2022 survey. (D) Proportion of individuals with anti-S antibodies in the February-March 2022 survey. Error bars indicate 95% confidence intervals. Anti-N: anti-nucleocapsid; anti-S: anti-spike antibodies.


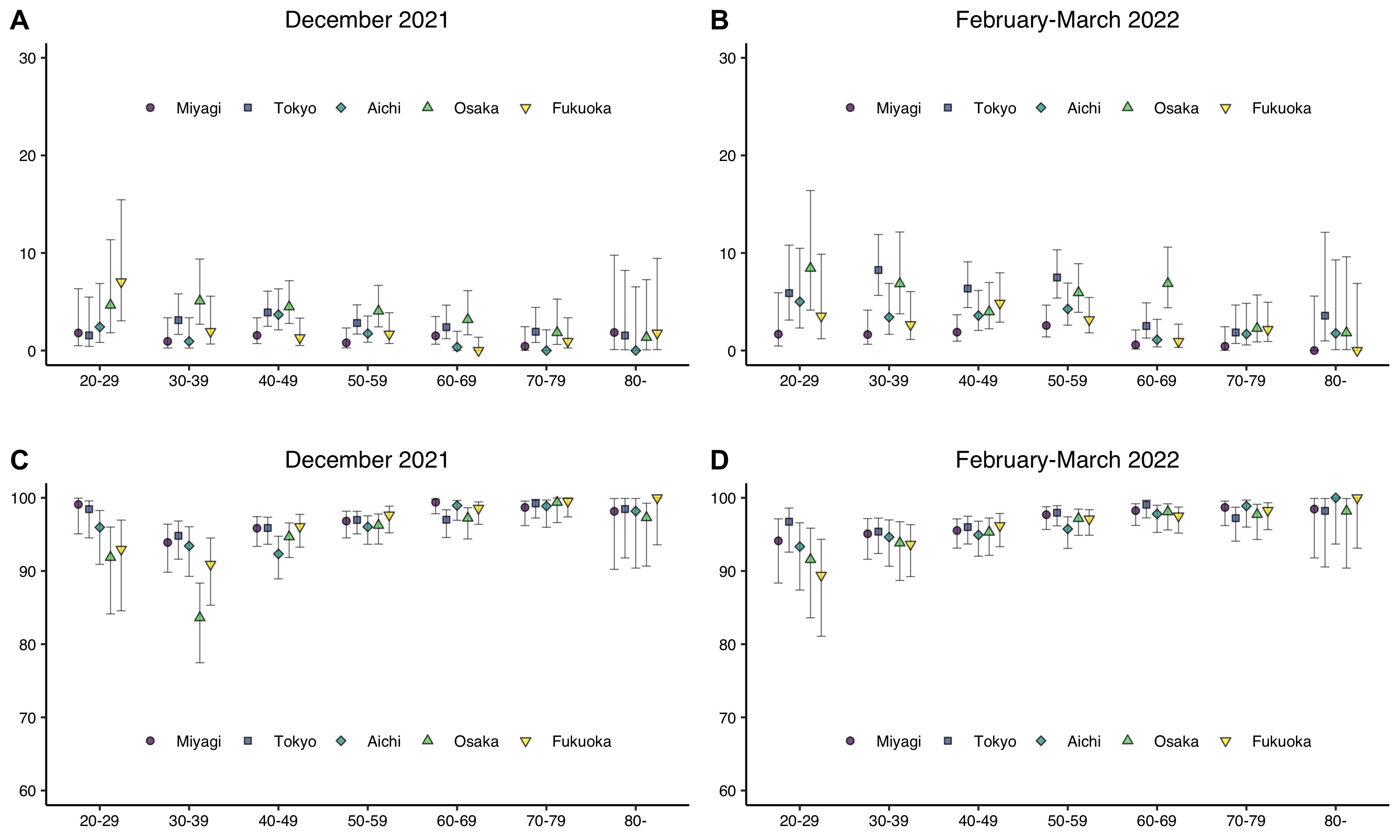

Supplement: Supplementary file 1 — Table S1. Proportion of population with individuals who reported to have past SARS‐CoV‐2 infection in the study population and cumulative proportion of reported COVID‐19 cases per 100 population in Japan by prefecture and age group Table S2. Crude seroprevalence and seroprevalence weighted by age group Table S3. The infection‐induced seroprevalence among individuals with a past diagnosis history of SARS‐CoV‐2 infection Figure S1. Sampling frame. Figure S2. Proportions of individuals with infection‐induced and vaccine/infection‐induced antibodies by age and prefecture. (A) Proportion of individuals with anti‐N antibodies (infection‐induced antibodies) in the December 2021 survey. (B) Proportion of individuals with anti‐S antibodies (infection/vaccination‐induced antibodies) in the December 2021 survey. (C) Proportion of individuals with anti‐N antibodies in the February–March 2022 survey. (D) Proportion of individuals with anti‐S antibodies in the February–March 2022 survey. Error bars indicate 95% confidence intervals. Anti‐N: anti‐nucleocapsid; anti‐S: anti‐spike antibodies. [file IRV-17-e13094-s001.docx]
